# General Notices

**Published:** 1893-08

**Authors:** 


### GENERAL NOTES.

By a thorough chemical test, the Warsaw Salt Baths have been pronounced as the strongest natural salt water baths in this country. This fact kept prominent before the readers of this journal is all that is essential in order to satisfy people who are in need of such treatment, where to go. The Sanitarium, located at Warsaw, N. Y., is one of the best in the country.

The Lambert Pharmacal Company, of St. Louis, Mo., may well lay great stress upon the real value of their preparation, which is fast becoming popular. Listerine is a preparation that can be used quite generally. It is becoming largely used by dentists all over the country. One dentist says, "The more I use Listerine the better I like it, and this I report after prolonged trial." Hundreds of such sentiments are sent to the manufacturers.

The existence under one roof of the schools of music, elocution, languages and fine arts, controlled by the one idea of the greatest efficiency at the lowest possible cost, makes the New England Conservatory of Music at once comprehensive and economical. It has, moreover, a most elegant and well appointed home, in which reside nearly four hundred lady students, under the care of an efficient preceptress and a lady physician of high reputation. The advantages of living, and taking all studies, and attending the excellent free courses of lectures, concerts, etc., all under one roof, is of immense importance to the student, as it does away with so many causes for loss of lessons, etc.

The Australian Pill receives hearty commendation from every source where it has had a fair trial. Write Dr. Worst of your ails and be convinced.

Horsford's Acid Phosphate needs no particular introduction to the readers of this journal. Its popularity has been established by its medicinal qualities.

It is claimed that what all the world is seeking, a successful treatment of consumption, is being carried on at the Sterlingworth Sanitarium, at Lakewood, New York. The institution publishes no circulars, but gives the results of the treatment, showing that they are curing consumption, although they do not claim to cure all victims of the disease.

Their original claim that climatic influence is not necessary for a cure will attract many who are banished to Colorado or similar climates in order to prolong life. The company invite the closest scrutiny, and will send abstract of cases already treated and furnish all the references asked in regard to patients who have already been cured.

We consider it our duty each month to call the attention of the lady readers of our journal to the *only* baking powder manufactured. It's the Royal.

One of the most delightful spots in this section of the country is the Delaware Water Gap. Healthful, picturesque and attractive. The Water Gap Sanitarium is an institution that has an open door for the feeble and sickly the year around, and is fast reaching a great popularity for its thorough treatment.

